# Genetic Markers PLEKHA7, ABCC5, and KALRN Are Not Associated With the Progression of Primary Angle Closure Glaucoma (PACG) in Malays

**DOI:** 10.7759/cureus.18823

**Published:** 2021-10-16

**Authors:** Lathalakshmi Thangavelu, Sarah Murniati Che Mat Nor, Darwish Abd Aziz, Sarina Sulong, Aung Tin, Liza Sharmini Ahmad Tajudin

**Affiliations:** 1 Department of Ophthalmology & Visual Science, School of Medical Sciences, Universiti Sains Malaysia, Health Campus, Kelantan, MYS; 2 Human Genome Centre, School of Medical Sciences, Universiti Sains Malaysia, Health Campus, Kelantan, MYS; 3 Department of Ophthalmology, Yong Loo Lin School of Medicine, National University of Singapore, Singapore, SGP

**Keywords:** malay, kalrn, abcc5, plekha, pacg

## Abstract

Introduction

*PLEKHA7*, *ABCC5,* and *KALRN* have been identified as susceptible genetic markers related to glaucoma. We aimed to investigate the association between the identified susceptible genetic markers PLEKHA7 rs11024102, ABCC5 rs17217796, and KALRN rs1392912 in the progression of primary angle-closure glaucoma (PACG) in Malay patients.

Methods

For this study, 163 Malay patients with PACG were recruited from April 2015 to April 2017 at Hospital Universiti Sains Malaysia and Hospital Raja Perempuan Zainab II, Kota Bharu. Venesection was performed. DNA was extracted using a commercial DNA extraction kit. The primer was optimized for rs11024102, rs17217796, and rs1392912 of the *PLEKHA7*, *ABCC5,* and *KALRN* genes, respectively. Polymerase chain reaction (PCR) was performed, and PCR products were purified. A DNA sequencer was used to identify polymorphisms. Progression was based on the agreement between the Advanced Glaucoma Intervention Study scoring system and the Hodapp-Parrish and Anderson staging system. The scoring was conducted on two reliable consecutive Humphrey visual fields (HVFs) during the recruitment period and two baseline HVFs obtained when the diagnosis was made. Based on the scoring, patients were grouped into progressed and non-progressed. A chi-square test was used to analyze the association between the genetic markers and the progression of PACG.

Results

One hundred and sixty-three Malay patients with PACG (58 men and 105 women) were recruited. Twenty-nine patients (18%) had visual field progression of PACG after a mean (SD) follow-up of 6.0 (1.0) years. The minor allele frequencies for PLEKHA7 rs11024102 (G/A), ABCC5 rs17217796 (C/G), and KALRN rs1392912 (A/G) were 0.44, 0.08, and 0.48, respectively. We found that rs11024102 (p=0.828), rs17217796 (p=0.865), and rs1392912 (p=0.684) were not associated with PACG progression in the Malay patients.

Conclusion

Although *PLEKHA7* and *ABCC5* were found to be genetic markers associated with the risk of PACG, they played no roles in PACG progression in the Malay population. Moreover, *KALRN* was not significantly associated with PACG progression. Other susceptible genetic markers may be responsible for PACG progression.

## Introduction

Glaucoma is a serious vision-threatening disease and a major cause of irreversible blindness in Asian populations [1-2]. Sixty percent of glaucoma cases in the world were reported to be from Asia with a prevalence of primary angle-closure glaucoma (PACG) of 0.73% in the entire continent [3-5]. Primary angle-closure glaucoma (PACG) is defined as primary angle closure (PAC) with evidence of glaucomatous optic nerve head damage with a corresponding visual field defect [6].

Glaucoma is a complex multifactorial disease possibly influenced by environmental and genetic factors. Through advances in molecular genetics and genome sequencing, multiple genetic loci contributing to glaucoma have been identified [7]. The genetic research on PACG in the Asian population is still in its infancy. However, several susceptible genetic markers have been identified by collaborative research involving 24 countries, including Malaysia [8-11].

The prevalence of PACG is higher in Asia than in Europe [12]. PACG in the Asian population was also found to behave differently from that in Caucasians [1,13]. PACG in the Asian population tends to behave like chronic asymptomatic glaucoma with a low percentage of acute angle closure (AAC) [14]. Many Asian patients with PACG are asymptomatic and have no history of AAC [15]. Laser peripheral iridotomy (LPI), the mainstay of treatment for AAC, has not conferred a protective effect against PACG progression [16-17]. However, most Caucasian patients with PACG showed no progression after LPI [18]. Therefore, the pathogenesis of PACG in Asians is believed to be more complex than that in Caucasians [19].

The Asian population is heterogeneous and comprised of multiple ethnicities, including Chinese, Indians, Japanese, and Malays. Asians have distinct features and most likely have different genetic compositions [20]. Most studies on PACG were focused on the Chinese because of the higher prevalence of PACG in this ethnic group. Malays contribute to 5% of the total world population. According to the Singapore Malay Eye Study, the prevalence of PACG was 0.12% [21].

In addition, retrospective studies have reported a higher progression rate in Malays [22-23]. This could be due to the late presentation and poor public awareness of PACG. However, the potential role of genetic variations in PACG progression could not be excluded. Identifying the risk factors of PACG progression is important in developing strategies to prevent blindness in the Asian region. No significant association was found between the rs3753841 (COL11A1), rs1015213 (PCMTD1-ST18), and rs11024102 (PLEKHA7) genetic markers and PACG progression in Chinese patients in Singapore [11]. The COL11A1 gene has been implicated in the regulation of aqueous humour production [24]. The PCMTD1 gene encodes protein-l-isoaspartate O-methyltransferase domain-containing protein 1, but its function is largely unknown. The ST-18 gene is known to be a mediator of apoptosis and inflammation [11,25]. The PLEKHA7 gene, which encodes pleckstrin homology domain-containing protein 7, is involved in paracellular permeability [26-27]. However, there is no report on the role of the PLEKHA 7 gene in the progression of PACG in Malays.

ABCC5 rs17217796 expression is also shown to have a significant association with the development of PACG, as it strongly contributes to anterior chamber depth [10]. The KALRN gene (rs1392912) was identified to be a potential susceptibility genetic marker of primary open-angle glaucoma (POAG) progression in Malays [28]. It plays an important role in the Rho-associated protein kinase (ROCK) pathway involved in trabecular meshwork activities [28]. As the occurrence of PACG is closely related to trabecular meshwork activities, the KALRN gene may also affect the risk of PACG progression. The genetic polymorphisms of these three genes may lead to aberrant fluid dynamics and trigger the onset of angle-closure [11]. Aberrant fluid dynamics in susceptible individuals may potentially accelerate the disease, causing further progression of the disease. Based on the potential role of these genetic markers, we selected them as candidate markers for the progression of PACG in Malay patients. The main aim of our study was to determine the association of selected genetic markers, namely, PLEKHA7 rs11024102, ABCC5 rs17217796, and KALRN rs1392912, with PACG progression.

## Materials and methods

Subjects

A cross-sectional study was conducted on 163 Malay patients with a confirmed diagnosis of PACG. The patients were recruited from two main tertiary hospitals in Kelantan, Malaysia, namely, Hospital Universiti Sains Malaysia and Hospital Raja Perempuan Zainab II, between 2015 and 2017. This study received ethical approval from the research and ethics committee of the School of Medical Sciences, Universiti Sains Malaysia, and Hospital Raja Perempuan Zainab II. The study was conducted in accordance with the principles stipulated in the Declaration of Helsinki.

Patients with underlying retinal, neuro-ophthalmology, or other systemic neurological diseases that interfere with visual field interpretation were excluded. Those with dementia and chronic or persistent disorders of the mental processes, brain disease or injury, memory disorder, and psychotic instability were also excluded. Only those who were compliant to follow-up, with a minimum of 18 outpatient visits, and achieved target pressure were included in this study. A pedigree chart was also drawn for at least three generations to exclude any interracial marriage. Patients with incomplete or doubtful pedigree charts were also excluded. Malays were defined as Malaysian citizens born to a Malaysian citizen who professes to be Muslim, habitually speak the Malay language, adhere to Malay customs, and are domiciled in Malaysia [29]. PACG was diagnosed on the basis of the International Society of Geographical and Epidemiological Ophthalmology [6]. PACG is defined as a PAC with evidence of glaucomatous optic nerve head damage with a corresponding visual field defect [8]. PAC is defined as an eye with an occludable drainage angle of ≥180° and features that indicate trabecular obstruction by the peripheral iris in the absence of glaucomatous optic disc damage.

Demographic data, including age at presentation, disease duration of glaucoma, and systemic comorbidities, were extracted from the patients' records in glaucoma clinics. Only one eye was included in this study; the eye with the worst PACG was chosen, or if both eyes have equally severe PACG and show progression, the right eye was chosen by default. The eye was thoroughly examined, including measurement of intraocular pressure (IOP) with the Goldman applanation tonometer (Haag Streit Bern, Switzerland) and visualization of the angle structure using a two-mirror gonioscopic lens. The vertical cup-to-disc ratio was determined clinically using a 78-diopter lens.

Two consecutive, reproducible, and reliable Humphrey visual fields (HVFs; Carl Zeiss, Dublin, CA) 24-2 analyses were used during the recruitment period. The HVF at baseline was obtained from the patient’s medical record. Only patients with two reliable and reproducible HVFs obtained within six months when the initial diagnosis was made were included in this study. A reliable HVF was based on fixation losses of <20% with false positives and negatives of <33%. Staging and scoring were conducted by the primary investigator (LT) and glaucoma consultant (LSAT) on four HVFs; two at baseline and another two in the recruitment period. The definition of progression was based on the agreement of two event-based analyses: Advanced Glaucoma Intervention Study (AGIS) scoring system and Hodapp-Parrish and Anderson (HPA) staging system [30-31]. Visual field progression is defined as the presence of change in a severity-based HPA staging system and worsening of 4 units or more based on the AGIS score. The progression of PACG was based on the agreement between the two investigators. Subsequently, patients with PACG were divided into the progressed and non-progressed groups.

Genotyping

Venesection was performed, and 6 ml of blood was collected into two ethylenediaminetetraacetic acid (EDTA) bottles. Deoxyribose nucleic acid (DNA) was extracted using the QIAGEN QIAamp DNA extraction kit (QIAGEN, Hilden, Germany) in accordance with the manufacturer's instructions. The concentration and purity of the extracted DNA were determined quantitatively by using a spectrophotometer (Biophotometer, Eppendorf, Germany). The double-stranded DNA concentration was measured at an absorbance wavelength of 260 nm. Blanking was performed by loading 50 μl of acetate/EDTA buffer (QIAGEN) into a disposable cuvette and spectrophotometry machine for measurement. Purity was determined by calculating the ratio of the A260/A280 wavelength, and the DNA purity ranged from 1.6 to 1.9. Only samples with a 260:280 ratio between 1.6 and 1.9 were used for further genetic analysis.

Three susceptible genetic markers, namely, rs1392912 (KALRN), rs17217796 (ABCC5), and rs11024102 (PLEKHA7), were chosen [10,20,28]. The Ensembl database was used for designing forward and reverse primers for the selected genetic markers (Table 1).

**Table 1:** Forward and reverse primers for rs11024102, rs17217796, and rs1392912 SNP, single nucleotide polymorphism; Ta, annealing temperature

Gene	SNP	Forward Primer	Reverse Primer	Ta	PCR product size (bp)
PLEKHA7	rs 11024102	CCTTTCCGA GGTCAAAGTCA	ACCAGAGC TTCCACCATG	56	611
ABCC5	rs 17217796	TCTCAGGTA GAACCCTGATG	CCATTCCTT ACACCACACTG	57	411
KALRN	rs 1392912	GTTCAAGCATTGCCTCTTCC	GGAGAAAACAGGCAGTGGTG	58	568

Polymerase chain reaction (PCR) was performed using Thermocycler SureCycler 8800 (Agilent Technologies, Santa Clara, CA). The reaction mixture for a single PCR amplification contains a PCR master mix (Thermo Scientific, Waltham, MA) of 12.0 μl, primer set of 2 μl (0.2 µmol each), and template DNA of 1 μl. A total reaction volume of 17 μl was used for PCR amplification. The PCR condition was as follows: initial denaturation at 95°C for 25 seconds, followed by 30 cycles of denaturation at 95°C for 25 seconds, and a specific annealing temperature of 56°C for rs11024102, 57°C for rs17217796, and 58°C for rs1392912. Elongation was performed at a temperature of 72°C for one minute; and the final extension at 72°C for five minutes.

The presence of amplicons was detected using 1% gel electrophoresis. PCR products were purified using the Illustra Exostar PCR product purification kit (GE Healthcare Bio-Sciences, Boston, MA). One microliter of PCR product, 1 μl of restriction enzyme, and 13.5 μl of purified water were incubated at 37°C for 15 minutes and further inactivated by heating at 80°C for 15 minutes in the thermal cycler. Finally, the purified PCR products were sent to a private laboratory (1st Base Laboratories, Selangor, Malaysia) for cycle sequencing in which dry ice was used to maintain the temperature. The result was interpreted using the BioEdit sequence alignment software (version 7.2.5).

Statistical analyses

Statistical analyses were performed using SPSS version 22 (IBM Corp, Armonk, NY). P < 0.05 was considered statistically significant. The chi-square test was used to compare the susceptible genetic markers between the non-progression and progression cases.

## Results

General characteristics

A total of 163 Malay patients with PACG (58 men and 105 women) were included in this study. Their mean (SD) age was 65.3 (1.5) years and the follow-up duration was 6.0 (1.0) years. The mean (SD) IOP on recruitment was 19.6 (3.0) mmHg. Forty-nine patients had a history of an acute angle-closure attack. Most patients (127) remained phakic, whereas 36 patients underwent cataract surgery. Twenty-four (24) patients had a history of trabeculectomy (14.7%). Systemic hypertension was the most common systemic comorbidity (71 patients, 43.6%), followed by hyperlipidemia (52 patients, 31.9%) and diabetes mellitus (46 patients, 28.2%). A total of 29 eyes were found to have visual field progression. No significant differences in sex (p = 0.573) and age (p = 0.789) were found between patients with and those without visual field progression (Table 2). However, eyes with visual field progression had longer follow-up durations (Table 2). A history of acute presentation of angle closure was less frequent in eyes with than in those without visual field progression (Table 2).

**Table 2:** Comparison of clinical parameters between the non-progress and progress groups SD, standard deviation; IOP, intraocular pressure; DM, diabetes; HPL, hyperlipidemia; APAC, acute primary angle closure; VCDR, vertical cup disc ratio; MD, mean deviation; PSD, pattern standard deviation; HPT, hypertension *p < 0.05 is considered statistically significance based on independent T-test. Tests for categorical data were performed using Pearson’s x2 test.

Variables	Non-progress	Progress	p-value
	N=134	N=29	
Gender (n, %)			
Male	49 (36.6)	9 (31.0)	0.573
Female	85 (63.4)	20 (69.0)	
Glaucoma Medication (n, %)			
Monotherapy	8 (6.0)	1(3.4)	0.841
Dual therapy	37 (27.6)	8 (27.6)	
Triple therapy	55 (41.0)	14 (48.3)	
Quadruplet therapy	13 (9.7)	2 (6.9)	
No treatment	21 (15.7)	4 (13.8)	
DM (n, %)	36 (26.9)	10 (34.4)	0.743
HPL (n, %)	37 (27.6)	15 (51.7)	0.05
HPT (n, %)	46 (34.3)	26 (89.7)	<0.001
APAC (n, %)			
Yes	38 (28.3)	11 (38.0)	0.308
No	96 (71.7)	18 (62.0)	
Mean age (mean, SD)	60.93 (8.73)	61.38 (5.57)	0.789*
VCDR (mean, SD)	6.69 (2.07)	7.62 (2.66)	0.041*
Mean MD (mean, SD)	-13.70 (8.14)	-18.8 (8.91)	0.003*
Mean PSD (mean, SD)	6.99 (3.65)	6.82 (2.93)	0.780*
Duration of disease (mean, SD)	5.01 (3.03)	7.10 (2.78)	0.001*

Genotype and allele frequencies of rs1392912, rs17217796, and rs11024102

The genotypes and allele frequencies of rs1392912, rs17217796, and rs11024102 are shown in Table 3.

**Table 3:** Comparison of genotype and allele frequency between the non-progress and progress groups MAF, minor allele frequency *p < 0.05 is considered statistical significance based on Fisher’s exact test. MAF was calculated using the Hardy-Weinberg equation (allele frequency = number of copies of allele in patients ÷ total number of all alleles for that gene in patients).

Genotype frequency (Genotype SNP)		Non-progress n (%)	Progress n (%)	p-value
KALRN				
GG		36 (26.9)	6 (20.7)	0.684
GA		67 (50)	17 (58.6)	
AA		31 (23.1)	6 (20.7)	
ABCC5				
GG		112 (83.6)	25 (86.2)	0.865
GC		21 (15.7)	4 (13.8)	
CC		1 (0.7)	0 (0)	
PLEKHA				
GG		30 (22.4)	5 (18.5)	0.828
GA		60 (44.8)	14 (48.1)	
A		44 (34.5)	10 (33.3)	
Allele frequency				
(SNP)	MAF			
KALRN				
G		39 (29.1)	8 (27.6)	0.620
A	0.48	95 (70.9)	21 (70.9)	
ABCC5				
G		112 (99.2)	25 (86.0)	0.726
C	0.08	22 (0.8)	4 (13.8)	
PLEKHA				
G	0.44	61 (45.5)	14 (48.3)	0.787
A		73 (54.5)	15 (51.7)	

**Figure 1:**
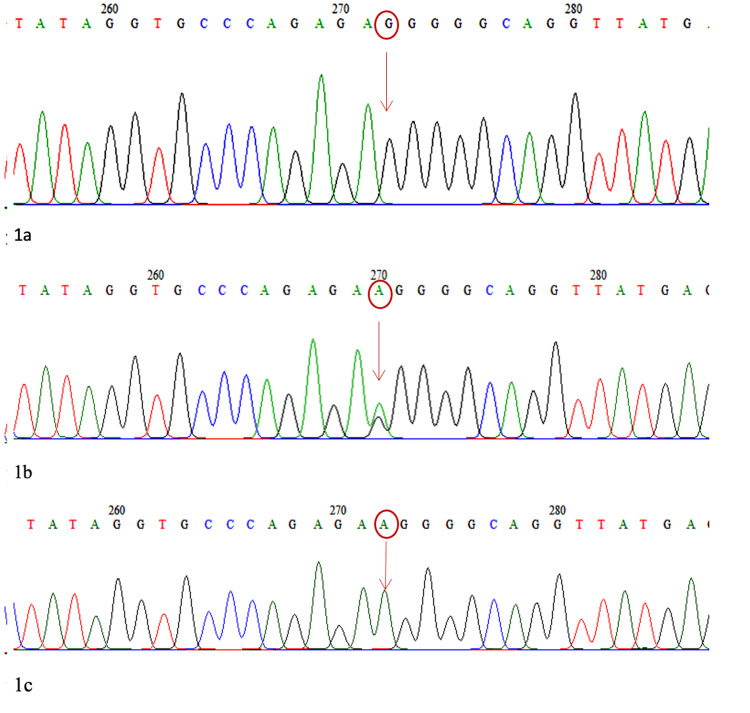
Electrochromatogram for KALRN 1a: Homozygous (GG) wild type, 1b: Heterozygous (GA), 1c: Homozygous (AA) mutant type

**Figure 2:**
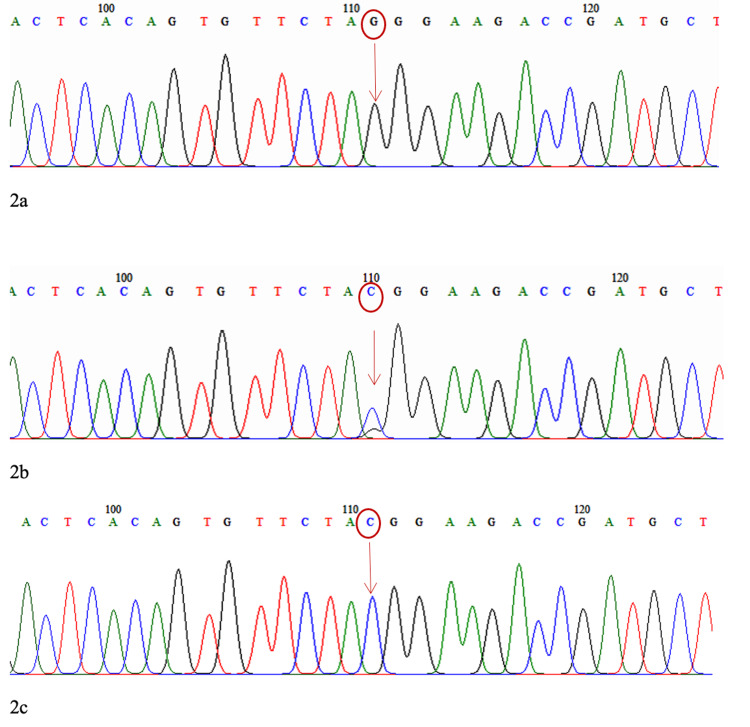
Electrochromatogram for ABCC5 2a: Homozygous (GG) wild type, 2b: Heterozygous (GC), 2c: Homozygous (CC) mutant type

**Figure 3:**
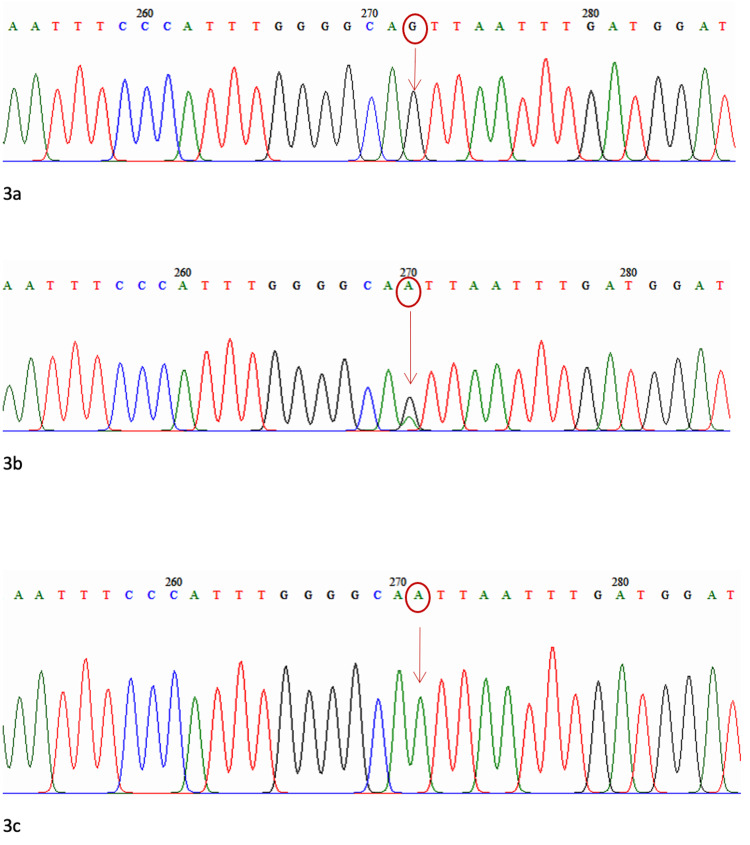
Electrochromatogram for PLEKHA7 3a: Homozygous (GG) mutant type, 3b: Heterozygous (GA), 3c: Homozygous (AA) wild type

The minor allele frequencies for PLEKHA7 rs11024102 (G/A), ABCC5 rs17217796 (C/G), and KALRN rs1392912 (A/G) were 0.44, 0.08 and 0.48, respectively. The sequencing results of the electrochromatograms for rs1392912, rs17217796, and rs11024102 are shown in Figures 1-3.

SNP rs1392912 is located at chromosome position 124647815 in the KALRN gene, SNP rs17217796 is located at chromosome position 183960606 in the ABCC5 gene, and SNP rs11024102 is located at chromosome position 16987058 in the PLEKHA7 gene. There was no significant difference in genotype and allele frequencies of rs1392912, rs17217796, and rs11024102 between the patients with and those without visual field progression.

## Discussion

In Asians, PACG is reported to cause more blindness than POAG and tends to progress faster in Asians [32]. Understanding the factors that affect PACG progression in Asians is important for effective management and planning preventive strategies against blindness. Genetics may be the reason for the predilection and different presentations of certain races towards developing PACG.

Various collaborative efforts have been made to identify potential genetic markers responsible for PACG [20,33]. So far, eight potential genetic markers, namely, rs11024102 PLEKHA7, rs1015213 PCMTD1-ST18, rs3753841 COL11A1, rs7494379 FERMT2 (PLEKHC1), rs1258267 CHAT (C10orf53), rs3739821 DPM2-FAM102A, rs736893 GLIS3, and rs3816415 EPDR1, were found to be significantly associated with susceptibility to the development of PACG in various populations [34]. Currently, many replication studies have been conducted in various other populations [8-9,35].

Most potential susceptible genes for PACG were involved with collagen synthesis and function, which may possibly explain the changes of the angle structure [33]. PLEKHA7 and ABCC5 gene expressions were found in structures relevant to glaucoma such as the iris, ciliary body, choroid, and aqueous humour outflow [10,20,36]. The PLEKHA7 gene encodes pleckstrin homology domain-containing protein 7, which is responsible for the maintenance and stability of the adherens junction between epithelial cells [26-27]. ABCC5 is also known as multidrug resistance protein 5, which has been shown to participate in tissue defense and cellular signal transduction mechanisms [37]. The single nucleotide polymorphisms (SNPs) of rs11024102 and rs1401999 of the PLEKHA7 and ABCC5 genes, respectively, were found to increase susceptibility to PACG [10,20].

KALRN expression was found to be involved in the Rho guanosine triphosphatase (GTPase) signal transduction pathway. Rho GTPase is a family of small G proteins that regulates a wide range of cellular activities, including cell proliferation, migration, adhesion, cytoskeletal dynamics, oncogenic transformation, inflammatory responses [38-40], and vascular smooth muscle contraction, by stimulating the downstream signaling of the ROCK pathway [41-42]. KALRN expression stimulates the ROCK pathway by activating a specific Rho GTPase (RhoA, RhoB, and RhoC) family member [43-44]. Increased expression and/or activity levels of RhoGEF (KALRN) proteins could augment contractile activation of the smooth muscles, which could reduce the supply of blood to the optic nerve head and result in glaucoma progression [28].

The KALRN gene was included in the present study. On the basis of a genetic study on Malay patients with POAG, rs1392912 and rs1660029 were found to increase the risk of susceptibility to POAG and progression of the disease [28]. The SNPs of rs1392912 and rs1660029 of KALRN were found to increase the risk of progression by 4.8-fold (odds ratio [OR], 4.76; 95% confidence interval [CI], 1.52-14.86) and 5.8-fold (OR, 5.88; 95% CI, 1.85-18.61), respectively. Owing to the potential racial influence, replicating this gene in Malay patients with PACG is justifiable.

Is there a role for these genetic markers in predicting the progression of PACG? Wei et al. found no association between these markers with PACG progression in Chinese patients in Singapore [11]. On the basis of racial variations, we replicated a similar study in Malay patients with PACG. However, the definition of progression in our study was more vigorous with the agreement of two event-based analyses, the AGIS and HPA staging systems. Only 29 eyes of 29 patients fulfilled the progression criteria.

No significant association was found between rs11024102 PLEKHA7, rs17217796 ABCC5, and rs1392912 KALRN and PACG progression in the present study. The main limitation of the present study is the small number of patients with PACG who developed HVF progression and the progression interval which is quite short. Perhaps, in the future, an equal number of patients in the progression and non-progression groups should be considered. The study was also a two-center study conducted in the same state, which may not truly represent the Malay population.

In this study, we used an event-based analysis by using the AGIS and HPA staging systems to identify progression. However, according to a few studies, an event-based analysis has its drawback of underestimating visual field progression compared with a trend-based analysis [45-46]. More patients with progression could have been identified if a trend-based analysis, such as mean deviation (MD) and visual field index (VFI), was used.

PACG is a complex disease with endophenotypes, including optic nerve head changes, IOP, and angle structure abnormalities. PACG progresses as a result of a combination of many factors, including compliance, adherence to treatment, and angle structures changes [47-48]. Only PACG patients who were compliant to follow-up visits and achieved target pressure were included. There are other factors that may influence the complexity of PACG. Ideally, to identify genetic influences, it is best if other confounding factors are controlled. A prospective cohort study is the best study design to evaluate PACG progression.

Including only one single ethnicity might have contributed to an overestimation of the genotype or allele frequency [49-50] in our study compared with studies that involved geographical ancestry [20,51]. Ethnicity and geographic ancestry are not interchangeable. These might have contributed to the negative findings of our study. The high degree of inbreeding effects in a small population or an isolated race results in an increased incidence of recessive disorders [52].

## Conclusions

In conclusion, rs11024102 (PLEKHA7), rs17217796 (ABCC5), and rs1392912 (KALRN) are not associated with PACG progression in Malay patients. The number of PACG patients who developed progression was rather small. Identification of genetic markers for susceptibility to PACG progression is important for the customization of treatment and the prevention of blindness in the future.
